# Supplementary material for: Negative Effect of Reduced NME1 Expression on Recurrence-Free Survival in Early Stage Non-Small Cell Lung Cancer
Source: J Clin Med. 2020 Sep 23;9(10):3067. doi: 10.3390/jcm9103067 (PMC7598190; doi:10.3390/jcm9103067)
Supplement: Supplementary file 1 [file jcm-09-03067-s001.pdf]

Table S1. Relationship between NME1 expression and clinicopathological characteristics (N=425).

Variables	NME1 expression		*P*-value
	Normal (N=260)	Reduced (N=165)	
Age[a]	61 ± 9	61 ± 10	0.93
Tumor size (cm)[a]	4.0 ± 2.1	4.1 ± 1.9	0.51
Pack-years (smoking)[a]	29 ± 27	32 ± 26	0.37
Sex			
Men	194	122	
Women	66	43	0.88
Smoking status			
Never	67	39	
Former	29	13	
Current	114	80	0.42
Pathologic stage			
IA	47	30	
IB	108	53	
IIA	54	44	
IIB	47	37	
IIIA	4	1	0.23
Histology			
Adenoca	130	65	
Squamous	108	92	
Others	22	8	0.01
Differentiation			
Well	45	29	
Moderate	115	79	
Poorly	36	31	
Undifferentiated	8	0	0.07
Adjuvant chemotherapy			
No	247	146	
Yes	13	19	0.01
Adjuvant radiotherapy			
No	236	146	
Yes	24	19	0.45
Recurrence			
No	176	63	
Yes	84	102	<0.0001

[a]Values indicate mean ± standard deviation. Abbreviations: Adenoca, adenocarcinoma; Squamous, squamous cell carcinoma.

Table S2. Univariate analysis of RFS (N=425).

Variables	HR[a]	95% CI	*p*-value
age	1.01	0.99-1.03	0.24
sex	1.22	0.89-1.67	0.21
Pack-years	0.99	0.98-1.01	0.26
Histology	0.80	0.63-1.03	0.08
Adjuvant chemotherapy	1.39	0.86-2.23	0.18
Differentiation	0.96	0.76-1.19	0.68
Pathologic stage	1.82	1.39-2.38	<0.0001
NME1	2.26	1.69-3.02	<0.0001
β-catenin	0.98	0.74-1.31	0.90

[a]Reference: patients with normal NME1 expression. Abbreviations: HR, hazard ratio; CI, confidence interval.

Table S3. Cox proportional hazards analysis[a] of RFS according to NME1 in.early-stage NSCLC (N=425), stratified by pathologic stages.

Pathologic stage	Reduced NME1 expression	HR[b]	95% CI	*p*-value
Stage IA (N=77)	Yes	3.93	1.76-8.74	0.0008
Stage IB (N=161)	Yes	2.52	1.49-4.29	0.0007
Stage IIA (N=98)	Yes	2.43	1.34-4.40	0.003
Stage IIB-IIIA (N=89)	Yes	1.64	0.93-2.89	0.08

[a]adjusted for age, sex, β-catenin expression, adjuvant chemotherapy, and histology; [b]Reference: patients with normal NME1 expression. Abbreviations: HR, hazard ratio; CI, confidence interval.

Table S4. Cox proportional hazards analysis[a] for RFS in early-stage NSCLCs (N=425).

Variables	HR	95% CI	*p*-value
NME1	2.27	1.70-3.03	<0.0001
Histology	0.64	0.49-0.83	0.0009
Pathologic stage	1.89	1.43-2.50	<0.0001

[a]adjusted for age, sex, β-catenin expression, and adjuvant chemotherapy. Abbreviations: HR, hazard ratio; CI, confidence interval.

Table S5. Cox proportional hazards analysis[a] for RFS according to NME1 expression in 425 early-stage NSCLCs treated with and without cisplatin-based adjuvant chemotherapy.

Adjuvant chemotherapy	Reduced NME1 expression	HR	95% CI	*p*-value
No (N=393)	No	1.00		
	Yes	2.19	1.61-2.96	< 0.0001
Yes (N=32)	No	1.00		
	Yes	3.26	1.16-9.17	0.03

[a]adjusted for age, sex, histology, β-catenin expression, and pathologic stage. Abbreviations: HR, hazard ratio; CI, confidence interval.

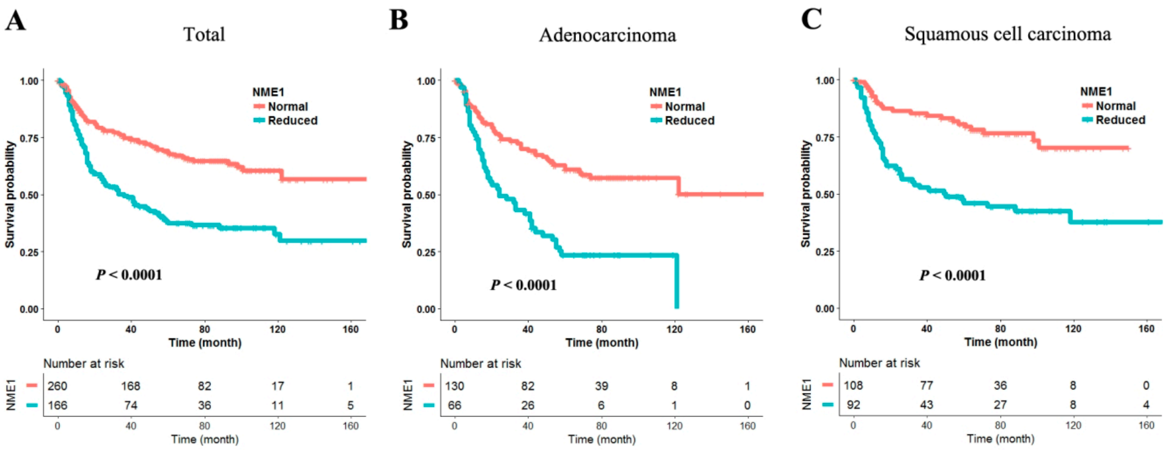

Figure S1. Kaplan-Meier plot of recurrence-free survival according to NME1 expression in histologic subtypes. The effect of reduced NME1 expression on RFS was analyzed using the log-rank test in 425 participants (A), 195 adenocarcinomas (B), and 200 squamous cell carcinomas (C).
